# Integrated Phytochemical and Pharmacological Investigation of *Mentha aquatica* L.: Anti‐Inflammatory, Analgesic, and Safety Evidence From In Vivo Studies

**DOI:** 10.1002/fsn3.72142

**Published:** 2026-07-31

**Authors:** Meryem Tourabi, Khaoula Faiz, Rafik EL‐Mernissi, Bouchra Louasté, Mohammed Merzouki, Karima El‐Yagoubi, Layla Tahiri Elousrouti, Abdel‐Rhman Z. Gaafar, Mulugeta Tesemma, Esmael M. Alyami, Ohoud A. Alghamdi, Musa A. Said, Hina Ali, Badiaa Lyoussi, Elhoussine Derwich

**Affiliations:** ^1^ Laboratory of Biotechnology, Conservation and Valorization of Bioresources, Faculty of Sciences Sidi Mohamed Ben Abdellah University Fez Morocco; ^2^ Laboratory of Biotechnology, Environment, Agri‐Food and Health, Faculty of Sciences, Dhar El Mahraz Sidi Mohamed Ben Abdellah University Fez Morocco; ^3^ Bioactives and Environmental Health Laboratory, Faculty of Sciences Moulay Ismail University Meknes Morocco; ^4^ Physiology and Physiopathology Team, Faculty of Sciences, Genomic of Human Pathologies Research Centre Mohammed V University Rabat Morocco; ^5^ Department of Pathology University Hospital Hassan 2 Fez Morocco; ^6^ Biomedical and Translational Research Laboratory, Faculty of Medicine and Pharmacy Sidi Mohamed Ben Abdellah University Fez Morocco; ^7^ Department of Botany and Microbiology, College of Science King Saud University Riyadh Saudi Arabia; ^8^ Department of Biology Bahir Dar University Bahir Dar Ethiopia; ^9^ Department of Biology, College of Science King Khalid University Abha Asir Saudi Arabia; ^10^ Research Centre for Advanced Materials Science (RCAMS) King Khalid University Abha Saudi Arabia; ^11^ Department of Chemistry, Faculty of Science Islamic University of Madinah Madinah Saudi Arabia; ^12^ Shanghai Key Laboratory for Molecular Engineering of Chiral Drugs, School of Chemistry and Chemical Engineering Shanghai Jiao Tong University Shanghai People's Republic of China; ^13^ Unity of GC/MS and GC‐FID, City of Innovation Sidi Mohamed Ben Abdellah University Fez Morocco

**Keywords:** acute toxicity, anti‐inflammatory activity analgesic activity, HPLC‐DAD, *Menta aquatica*, phytoconstituents, phytotherapeutic applications, subacute oral toxicity

## Abstract

This study investigated the chemical composition, safety profile, and pharmacological activities of a decocted extract from the aerial parts of *Mentha aquatica* (MA‐DE). High‐performance liquid chromatography with diode‐array detection (HPLC‐DAD) identified several phenolic constituents, mainly hydroxycinnamic and hydroxybenzoic acids. Safety was evaluated through acute and subacute toxicity studies in albino mice. Acute toxicity testing involved oral and intraperitoneal administration of MA‐DE at doses up to 8 g/kg body weight (BW). Oral administration produced no mortality or observable toxic effects, whereas intraperitoneal administration induced dose‐dependent toxicity, with an LD_50_ of 5.975 g/kg BW; the NOAEL and LOAEL were determined as 0.5 and 1 g/kg BW, respectively. In the subacute study, mice received daily oral doses of 0.1, 0.5, or 1 g/kg BW for 28 days, with no significant changes observed in hematological, biochemical, or histopathological parameters compared with controls. The anti‐inflammatory activity of MA‐DE was assessed using the carrageenan‐induced paw edema model in Wistar rats, while analgesic activity was evaluated using the writhing test. MA‐DE exhibited significant, dose‐dependent anti‐inflammatory and analgesic effects at 200 and 400 mg/kg BW. Overall, MA‐DE appears safe when administered orally and demonstrates promising pharmacological properties, supporting its traditional use and potential phytotherapeutic application.

## Graphical abstract

*Mentha aquatica* decocted extract contained mainly phenolic acids and showed a favorable oral safety profile in mice, with no significant subacute toxicity. Intraperitoneal administration caused dose‐dependent toxicity. The extract exhibited significant dose‐dependent anti‐inflammatory and analgesic activities in rodent models, supporting its potential as a safe, bioactive natural therapeutic.

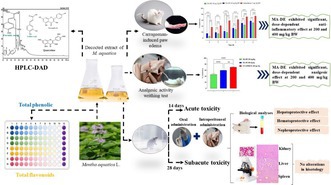

## 1 Introduction

Medicinal herbs have been consumed for a long time as a natural remedy to prevent and treat a range of illnesses due to their capacity to promote health (Kumar and Correspondence 2016; Sajjad et al. 2025). Their therapeutic benefits are largely attributed to the presence of bioactive compounds such as polyphenols, flavonoids, and essential oils (Veeresham 2012). According to the World Health Organization (WHO), a significant portion of the global population still relies on traditional medicine as part of primary healthcare (Che et al. 2017). This widespread reliance stems not only from cultural traditions but also from the increasing preference for natural substances, which are perceived to have fewer side effects compared to synthetic drugs (Kumar and Correspondence 2016). Traditional medical practices and herbal formulations have long been used in both developed and developing countries due to their minimal adverse effects and their origin from locally available natural resources (Süntar 2020). Moreover, before incorporating plant‐based products into modern therapeutic applications, it is essential to assess their safety through comprehensive toxicological evaluation. Regulatory authorities such as the U.S. Food and Drug Administration (FDA) require preclinical toxicity data to ensure the safety of compounds intended for human use. Acute and subacute toxicity studies in animal models remain the gold standard for establishing safety profiles of herbal extracts prior to clinical testing (Andrade et al. 2016; Arome and Chinedu 2013; Patel et al. 2024).

The genus *Mentha* is classified under the family Lamiaceae. It contains 25 to 30 species widely distributed in subtropical and tropical countries of both hemispheres (Salehi et al. 2018). These species are rich in phytoconstituents, including phenolic acids and flavonoids, which have been associated with various biological activities (Fialová et al. 2015). Among them, *Mentha aquatica* L., popularly called Water mint, is an annual plant that is native to Europe and is grown in North America, Europe, Asia, Australia, and South America (McKay and Blumberg 2006). Traditionally, it is largely used in folk medicine for its antispasmodic, analgesic, antimicrobial, and anti‐inflammatory properties. It has also been used to relieve digestive issues, colds, and toothaches. Several studies have demonstrated the pharmacological potential of *M. aquatica* extracts, highlighting activities such as antiviral, hepatoprotective, cytoprotective, and antiulcer effects (Truong 2022). In Morocco, decoction is the traditional extraction method used for this species. This approach closely replicates the conditions of traditional human use, thereby enhancing the ethnopharmacological relevance of the study. Previous research has found that the Venda people of South Africa use a combination of dried *M. aquatica* and *Tagetes minuta* L. leaves, which are burnt, to treat mental diseases (Arnold and Gulumian 1984) as a therapy for respiratory illness “colds”, and to keep off “curses” and “bad spirits”.

Previous phytochemical reports on *M. aquatica* have proved effective in identifying and characterizing a broad range of bioactive molecules with a large spectrum of biological effects, including phenolic acids (chlorogenic acid, p‐coumaric acid, benzoic acid, ferulic acid, caffeic acid, trans‐cinnamic acid), flavonoids (kaempferol, rutin, apigenin, naringenin‐7‐O‐rut, hesperetin‐7‐O‐rut, luteolin glucoside, quercetin), and terpenes (Pereira et al. 2019; Park et al. 2019; Dorman et al. 2003; Andro et al. 2013). Despite these findings, existing research has focused primarily on identifying these compounds and describing their pharmacological effects, with limited attention to the plant's safety profile. To the best of our knowledge, this study presents the first toxicological evaluation of a decocted extract of *M. aquatica* collected in Morocco. The objective is to unveil the phenolic content and profile, and to assess its acute and subacute oral toxicity in both male and female Swiss albino mice using experimental models, concerning single exposure (evaluating acute toxicity) or repeated exposure during 28 days (assessing subacute oral toxicity). This dual approach aims to establish a scientific basis for the safe traditional use of this plant and to support its integration into evidence‐based herbal medicine.

## 2 Materials and Methods

### 2.1 Collection of Plant Material

Aerial parts of *M. aquatica* were collected in the city of Merja Zerga (Moulay Bousselham lagoon), in the northern region of Morocco (06°18′10″W longitude and 34°52′30″N latitude), in April 2021. *M. aquatica* was identified by the Botanist Amina Bari, and the voucher specimen has been deposited in the Herbarium of the University Sidi Mohamed Ben Abdellah University, Fez, Morocco under 002MAMZ2121. After 2 weeks of shade‐drying, the plant's aerial parts were removed and ground into a powder using a high‐quality herb grinder. Until extraction, the powder was kept in a dark glass container.

### 2.2 Preparation of the Plant Formulation

The extraction was performed by the decoction process. Briefly, powdered *M. aquatica* (50 g) was mixed with distilled water (500 mL) at 40°C for 20 min. After boiling, the mixture was allowed to cool to room temperature and then filtered through a Whatman No. 1 filter solely to remove undissolved particles from the decocted extract. The filtrate was then concentrated using a rotary vacuum evaporator at 40°C (Büchi R‐210, Flawil, Switzerland). The solvent was removed to obtain crude extracts, and the obtained extracts were stored at −20°C until application. The crude extract obtained was dissolved in distilled water each day for toxicological assays in the present experiment.

### 2.3 HPLC‐DAD Analysis

Reverse‐phase High‐Performance Liquid Chromatography (HPLC) coupled with a Diode Array Detector (DAD) was used to examine the phytochemical content of the *M. aquatica* decocted extract. Before HPLC analysis using a SHIMADZU system, the extracts (10 mg) were filtered through 0.45 μm filters and diluted to 1 mL with 80% methanol. At 40°C, phenolic compounds were divided on a Wakosil C18HG column (5 μm, 4.6 × 150 mm). Utilizing a mixture of binary solvents of 50/50 methanol/acetonitrile (solvent A) and 0.2% phosphoric acid with water acidified (solvent B), the elution was executed in the gradient procedure. There was a linear gradient performed between 96% (A) and 4% (B) to 50% (A) and 50% (B) over 40 min following a 12‐min re‐equilibration to the original composition. Afterwards, it alternated between 40% (A) and 60% (B) for 5 min, and then between 0% (A) and 100% (B) for a total of 15 min. For the mobile phase, each component was injected at a flow rate of 1 mL/min and a volume of 20 μL. All phenolic chemicals (coumaric acid, vanillic acid, caffeic acid, syringic acid, gallic acid, pyrogallic acid, ferulic acid, rosmarinic acid, protocatechuic acid, tannic acid, rutin, quercetin, catechol, and kaempferol) were determined by considering the length of time they were retained with the retention time of standard compounds (Tourabi et al. 2023).

### 2.4 Quantification of the Total Phenolic Content of *M. aquatica* Decocted Extract

Using the Folin–Ciocalteu assay, the total phenolic content (TPC) of the aqueous extract at 2 mg/mL subsequently subjected to serial (cascade) dilutions was determined (Singleton and Rossi 1965). The standard deviation graph was prepared using gallic acid (0.016–1 mg/mL; *R* ^2^ = 0.9998) as a reference. The TPC data were presented as mg GAE/g DW.

### 2.5 Quantification of the Total Flavonoid Content of *M. aquatica* Decocted Extract

The aluminum trichloride (AlCl_3_) test was used to determine the total flavonoid concentration of the aqueous extract at 2 mg/mL subsequently subjected to serial (cascade) dilutions (Shraim et al. 2021). Quercetin was employed as a standard at concentrations ranging from 0.008 to 0.125 mg/mL to generate the standard curve (*R* ^2^ = 0.9949). The TFC results were presented as mg QE/g DW.

### 2.6 Animal Models

In the present experiment, mature Swiss albino mice, 8 weeks of age, both male and female, weighing 25–35 g, were used. The Swiss albino mice were kept in plastic cages for a week to adjust to the laboratory climate (T: 25°C, 12 h of light).

### 2.7 Experimental Design

#### 2.7.1 Acute Toxicity Assessment

The single intraperitoneal and oral acute toxicity of the decocted extract of *M. aquatica* was carried out following OECD Guideline No. 423 (OECD 2001). Eight groups of animals were established (*n* = 5 per sex/group) and fed for 4 days on solid food and potable water. The ultimate volume of analytical specimens was administered orally to the animals at the following ratios: 0.5 mL/20 g BW, and injected intraperitoneally at 0.4 mL/20 g BW (Tourabi et al. 2023). The *M. aquatica* decocted extract was precipitated in distilled water and injected intraperitoneally at doses of 0, 0.5, 1, 2, 4, and 8 g/kg BW, and orally at doses of 0, 0.5, 1, 2, 4, and 8 g/kg BW. Groups were observed daily for 14 days during the treatment period for global behavioral changes, danger signs, and death (Silva et al. 2007). The formulation described by Litchfield and Wilcoxon U was employed to calculate the lethal dosage (LD_50_) (Litchfield and Wilcoxon 1949).

#### 2.7.2 Subacute Oral Toxicity Assessment

The repeated‐dose oral toxicity assessment was conducted in accordance with OECD TG No. 407 for 28 consecutive days. Three groups of animals were randomly selected (*n* = 5 per sex per group). Animals were randomly allocated into experimental groups using a simple randomization procedure. The untreated group received standard saline solution. In contrast, the other groups that received treatment were given the *M. aquatica* decocted extract orally at low (0.1 g/kg bw), middle (0.5 g/kg bw), and high (1 g/kg bw) doses, respectively. For the period of the 28‐day treatments, body mass changes, harmful symptoms, and deaths were monitored every day. At the end of the experiment, mice were anesthetized, and blood samples were obtained via retroorbital sinus puncture for hematological and biochemical analysis (Waynforth and Flecknell 1995). Kidneys, livers, and spleens were carefully removed and weighed after being placed in a physiological solution of 0.9% sodium chloride (NaCl) in water.

### 2.8 Relative Organ Weights

The relative organ weight (ROW) of the liver, kidney, and spleen in animals receiving different treatments was determined using the equation provided by Chavalittumrong et al. (2004).

### 2.9 Determination of Hematological and Biochemical Markers

The whole blood was collected in heparinized tubes for hematological markers using the blood automated analyzer (Pentra 80XL); hemoglobin (HGB), Red blood cells (RBC), white blood cells (WBC), mean corpuscular hemoglobin (MCH), mean corpuscular hemoglobin concentration (MCHC), hematocrit (HCT), mean corpuscular volume (MCV), platelet count (PLT), and differential blood count (Neutrophils, eosinophils, basophils, lymphocytes, and monocytes). While plasma was separated by centrifugation at 4000 rpm for 10 min at 4°C to obtain serum for hepatic parameter analysis, which was then stored at −20°C until further measurements were conducted. Serum creatinine, urea, aspartate aminotransferase (AST, SGOT), and alanine aminotransferase (ALT, SGPT) have been measured using an automated chemical analyzer (Cobas 6000 and Integra 400).

### 2.10 Histological Examination in the Subacute Oral Toxicity

At the end of the experimentation, animals were euthanized (70 mg/kg Ketamine, 10 mg/kg Xylazine), before organs were collected, and the kidney, liver, and spleen weights were recorded to determine relative organ weight. The specimens were checked for any macroscopic abnormalities (Mobashar et al. 2025). All of these organs have been preserved in 10% formalin for histological investigation. The tissue sample was fixed in paraffin and then cut into 5‐μm‐thick slices for hematoxylin–eosin (H&E) staining. Histological alterations on prepared tissue slides were examined by a qualified pathologist using a light microscope. The observed lesions were assessed semi‐quantitatively using a scoring scale ranging from 0 (no lesion) to 4 (severe lesion), according to the criteria established by Shackelford et al. (2002). Histological assessments were performed in a blinded manner to avoid bias.

### 2.11 Anti‐Inflammatory Activity

#### 2.11.1 Carrageenan‐ Induced Rat Paw Edema Test

The current study employed carrageenan‐induced rat hind paw edema as an animal model of acute inflammation, as described by Zouhri et al. (2023). In short, 1 h after the test materials were given orally, 0.1 mL of carrageenan solution (1%) was subplantarly injected into the mice's right hind paw to cause acute inflammation. A plethysmometer (Panlab LE 7500) was used to measure the paw volume 1, 2, 3, 4, 5, and 6 h following the carrageenan injection. The doses of the extract were 150 mg/kg and 300 mg/kg body weight. The typical anti‐inflammatory drug used was 10 mg/kg body weight of indomethacin.

Equation (1) was used to determine the anti‐inflammatory efficacy as a percent inhibition:

(1) $$\text{Inhibition}\;\left(\%\right)=\frac{\left(\mathrm{VC}-\mathrm{VT}\right)}{\mathrm{VC}}\times 100$$

***Vc*** is the average increase in the paw volume of the control group, and ***Vt*** is the average increase in the paw volume of the treatment group.

### 2.12 Analgesic Activity

#### 2.12.1 Writhing Test

As previously stated by (Zouhri et al. 2023), acetic acid‐caused contortion was performed. The mice were weighed (25–30 g) and then separated into four groups of six mice each.

- Group 1 (control): Received or distilled water (1 mL/100 g bw).
- Group 2: Received aspirin orally (100 mg/kg bw).
- Group 3: Received orally ML‐extract (150 mg/kg bw).
- Group 4: Received orally ML‐extract (300 mg/kg bw).

After 30 min following the treatments, 3.75 mL/kg bw of intraperitoneal acetic acid solution (3%) was administered to cause distortions. After the injection of acetic acid, a 10‐min observation time was used to tally the contortion numbers. The following Equation (2) was applied to calculate the inhibition (%) of abdominal constrictions.

(2) $$\text{Inhibition}\%=\left(1-\left(\mathrm{Wt}/\mathrm{Wc}\right)\right)\times 100$$

where Wt and Wc represent the contortion numbers in the treated and control groups, respectively.

### 2.13 Statistical Analyses

The findings are shown as the median value's standard error of the mean (± SEM). Using GraphPad Software 8.0.1, a one‐way ANOVA and a subsequent Tukey test were used to determine statistical significance among the groups. Significance was determined at an average level that was less than 5% (*p* < 0.05).

## 3 Results

### 3.1 Identification and Quantification of Individual Phenolic Compounds of *M. aquatica* Decocted Extract Using HPLC‐DAD Analysis

**FIGURE 1:**
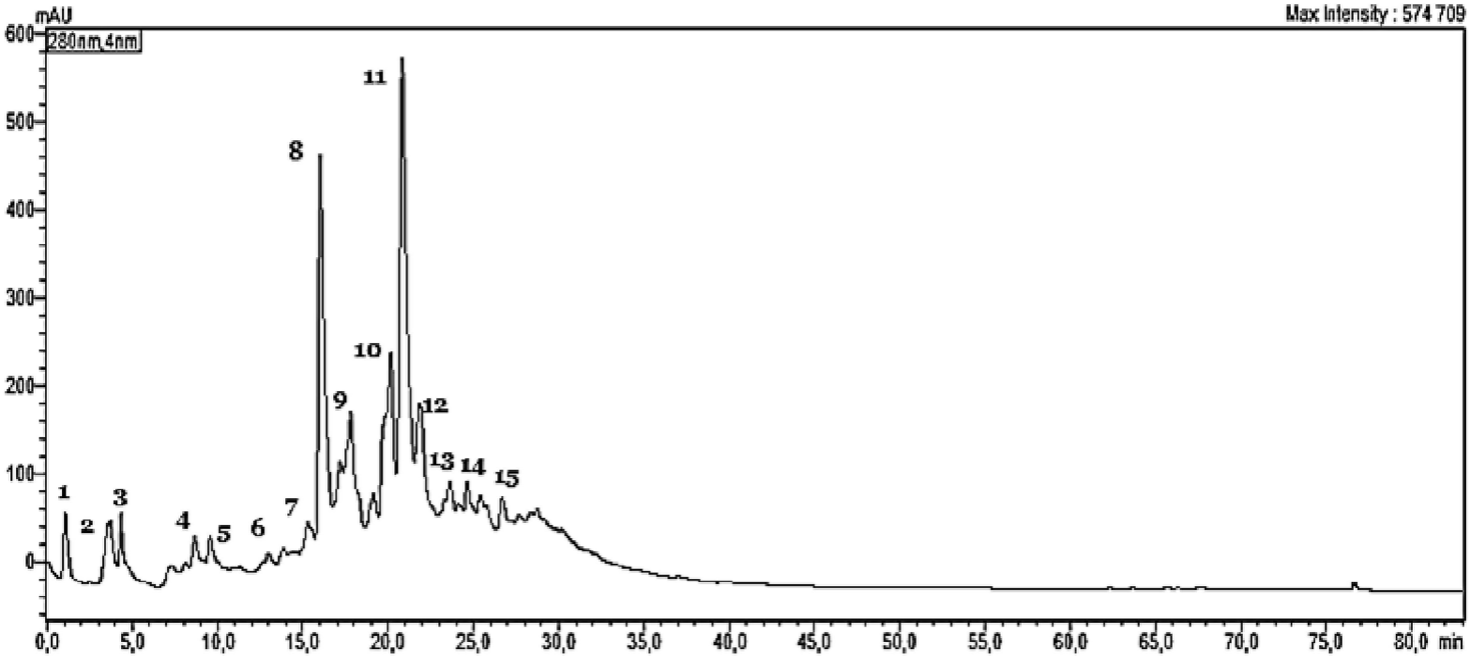
HPLC chromatogram of decocted extract of *M. aquatica* , tannic acid (peak 1), pyrogallol (peak 2), gallic acid (peak 3), caffeic acid (peak 4), kaempferol (peak 5), syringic acid (peak 6), coumaric acid (peak 7), ferulic acid (peak 8), vanillic acid (peak 9), rutin (peak 11), quercetin (peak 15).

The individual phenolic compounds of the decocted extract of *M. aquatica* are displayed in Table 1, and the result demonstrates that 11 phenolic compounds have been identified in this extract. The major molecules identified in our sample are rutin, ferulic acid, and vanillic acid (Figure 1). Similarly, the other phenolic compounds detected are pyrogallol, caffeic acid, gallic acid, coumaric acid, syringic acid, tannic acid, quercetin, and kaempferol.

**TABLE 1:** Identification and quantification of individual phenolic components in *M. aquatica* decocted extract.

Phenolic compounds	*M. aquatica*
	Concentration (mg/g extract)
	*Hydroxycinnamic acids*
Ferulic acid	10.73 ± 0.06
Caffeic acid	0.83 ± 0.04
Vanillic acid	7.10 ± 0.00
Coumaric acid	0.56 ± 0.23
Rosmarinic acid	ND
	*Hydroxybenzoic acids*
Syringic acid	0.28 ± 0.09
Protocatechuic acid	ND
Gallic acid	1.24 ± 0.15
Pyrogallic acid	1.20 ± 0.07
	*Flavonoids*
Rutin	14.89 ± 0.03
Quercetin	1.09 ± 0.11
Catechol	ND
Kaempferol	0.41 ± 0.01
	*Other*
Tannic acid	1.07 ± 0.03

Abbreviation: ND, non‐determined.

### 3.2 Determination of the Total Content of Flavonoids and Phenols

**FIGURE 2:**
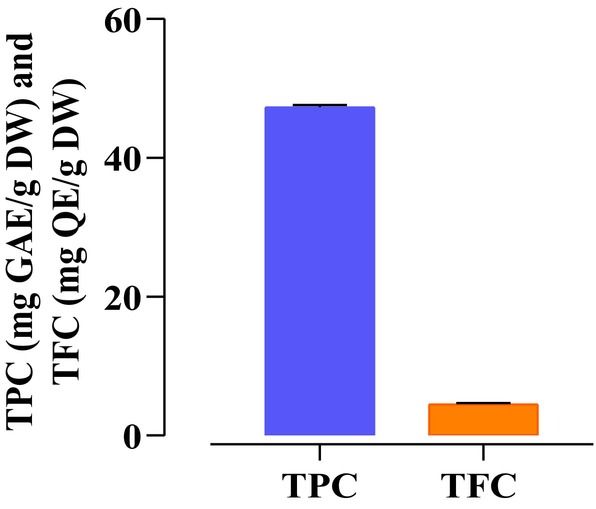
Total amounts of phenols and flavonoids in the decocted extract of *M. aquatica* ; results are expressed as the mean ± SD.

The data obtained in the assessment of the flavonoid and phenolic content of *M. aquatica* decocted extract are displayed in Figure 2. The Folin‐Ciocalteau assay was used to determine the total amount of phenol, while the flavonoid content was determined using the colorimetric method (AlCl_3_). The *M. aquatica* decocted extract exhibits a high polyphenol content, along with a notable flavonoid content.

### 3.3 Acute Toxicity Assessment of *M. aquatica* Decocted Extract in Mice Administered Orally and Intraperitoneally

Acute toxicity testing revealed that a single administration of *M. aquatica* decocted extract given orally at the tested doses (0.5, 1, 2, 4, or 8 g/kg BW) did not result in death or toxicological symptoms within 24 h to 14 days in either males or females. No signs of hypoactivity, piloerection, lethargy, convulsions, or mortality were observed in any of the mice (Table 2). On the other hand, the correlation between the doses and the death rate or acute toxicity of the *M. aquatica* decocted extract injected intraperitoneally enhanced gradually (Table 2). The lethality rate remained at 0% up to a dose of 4 g/kg BW, but gradually improved to 100% at the highest concentration tested, 8 g/kg BW. The approximate lethal dose (LD_50_) for intraperitoneal acute toxicity of decocted extract was 5.975 g/kg BW.

**TABLE 2:** Examination of the single dose (acute toxicity) of decocted extract from *M. aquatica* administered via gavage and intraperitoneal injection in mice.

Dose (g/kg BW)	Sex	Administration pathways					
		Oral			Intraperitoneal		
		D/T	Latency	Sign of toxicity observed	D/T	Latency	Sign of toxicity observed
Control	M	0/5	—	None	0/5	—	None
	F	0/5	—	None	0/5	—	None
0.5	M	0/5	—	None	0/5	—	None
	F	0/5	—	None	0/5	—	None
1	M	0/5	—	None	1/5	—	Hypoactivity, convulsions
	F	0/5	—	None	0/5	—	None
2	M	0/5	—	None	0/5	—	Hypoactivity, piloerection
	F	0/5	—	None	0/5	—	Hypoactivity
4	M	0/5	—	None	0/5	2 h	Hypoactivity, piloerection, Lethargy
	F	0/5	—	None	0/5	—	Syncope, asthenia, convulsions
8	M	0/5	—	None	5/5	24–48 h	Syncope, diarrhea, convulsions, and Lethargy
	F	0/5	—	None	5/5	24–48 h	Syncope, convulsions, piloerection

**FIGURE 3:**
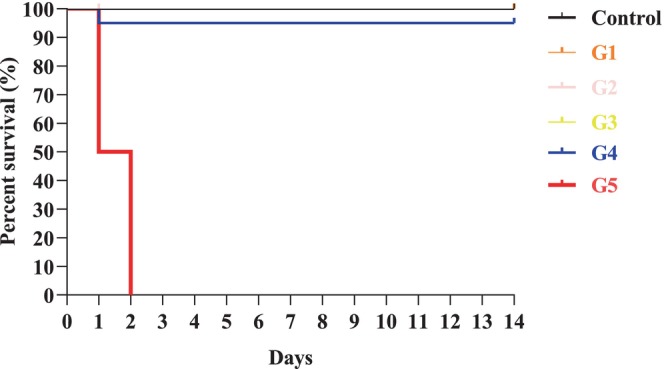
Survival plots for the intraperitoneal administration of *M. aquatica* decocted extract were observed in 5 groups over 14 consecutive days, alongside an untreated group (control). The animals are treated with various doses of decocted extract of *M. aquatica* , namely, Control group (0 g/kg BW); G1 (0.5 g/kg BW); G2 (1 g/kg BW); G3 (2 g/kg BW); G4 (4 g/kg BW); G5 (8 g/kg BW). The results are described as mean ± SEM (*n* = 5 animals/sex/group), with a significance level of *p* < 0.001.

The survival plot displays the survival probabilities of five groups: one that received the intraperitoneal administration of *M. aquatica* decocted extract G 1 (0.5 g/kg), G 2 (1 g/kg), G 3 (2 g/kg), G 4 (4 g/kg), G 5 (8 g/kg), and a control group that either received a saline solution (Figure 3). The indications, symptoms, intoxication duration, and mortality rate were analyzed. In a dose‐dependent process, the decocted extract of *M. aquatica* caused mortality in mice. The symptoms frequently commenced within 2 h after intraperitoneal injection, with immediate signs such as hypoactivity, piloerection, lethargy, dyspnea, and convulsions becoming evident. Mortality was mainly observed after 24 h, with mice that remained alive after this time frame surviving throughout the study. Following the injection of the decocted extract, cases of death were recorded within 1 day, as shown in Figure 3. Notably, the estimated lethal dosage (LD_50_) for the *M. aquatica* decocted extract in Swiss albino mice was determined to be 5.975 g/kg BW.

### 3.4 Subacute Oral Toxicity Evaluation of *M. aquatica* Decocted Extract in Mice

**FIGURE 4:**
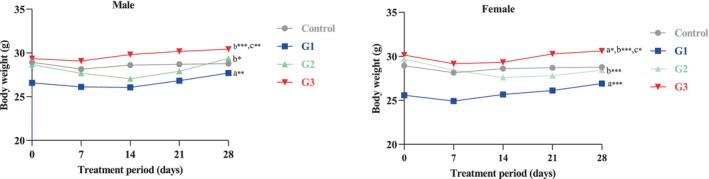
Body mass of both female and male in subacute oral toxicity experiment of the various doses of *M. aquatica* decocted extract, 0 mg/kg (Control), G1 (100 mg/kg), G2 (500 mg/kg), and G3 (1000 mg/kg bw). The obtained findings were presented as a mean ± S.E.M. (*n* = 5/sex/group, *p < 0.05; **p < 0.01; ***p < 0.001). (a) Define the distinction between control and dose (G1, G2, and G3); (b) Explain the distinction between G1 mg/kg and other groups (G2, G3); c Identify the differences (G2 and G3).

The findings indicated that mice administered daily oral doses of decocted extract from *M. aquatica* (100, 500, or 1000 mg/kg BW) for 28 consecutive days did not exhibit any mortality in either sex. Furthermore, an important modification in body weight at all observation periods is shown in Figure 4. There is a statistically significant variation in body mass of the treated groups (100–1000 mg/kg BW) in comparison to the untreated group (control) for both male and female mice (*p* < 0.01 and *p* < 0.001).

**FIGURE 5:**
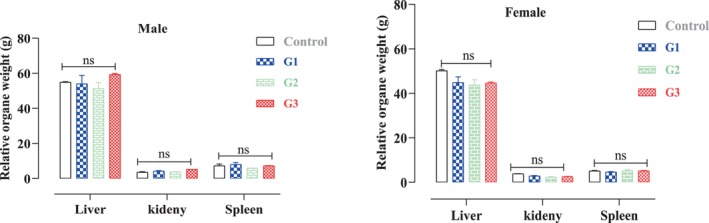
After administering *M. aquatica* decocted extract to male and female mice at different dosages of 0 mg/kg BW (Control), G1 (100 mg/kg BW), G2 (500 mg/kg BW), and G3 (1000 mg/kg BW) for 28 days, the relative (%) masses of their kidneys, livers, and spleens were calculated. The results are presented as mean ± SEM. Statistical analysis revealed no noticeable differences between the treated groups and the untreated group (control) (*p* > 0.05).

In addition, as illustrated in Figure 5, no substantial distinction was observed in the relative organ weight (ROW) of the liver, spleen, and kidneys between the treatment groups and the untreated control group through all dosage levels in both female and male Swiss mice (*p* > 0.05).

#### 3.4.1 Effect of Oral Administration of *M. aquatica* Decocted Extract on the Hematological and Hepatic Markers of Swiss Mice

The impact of the decocted extract of *M. aquatica* on the hematological and hepatic markers in the oral administration experiment is presented in Tables 3 and 4. In a subacute study, the mice were treated with the decocted extract of *M. aquatica* at 100, 500, and 1000 mg/kg BW for 28 days, and various biochemical parameters, notably AST, ALT, urea, and creatinine, were measured. As illustrated in Table 3, there was no significant alteration in the level of ALT, along with non‐significant variations in the level of ALT for the treated group, in contrast to the control group for male mice. Besides, the 100 mg/kg extract showed a slight decrease in AST levels, with non‐significant changes in ALT levels in male and female mice (*p* < 0.05 and *p* > 0.05, respectively).

**TABLE 3:** Biochemical parameters in mice orally exposed to *Mentha aquatica* leaf decocted extract.

Liver parameters	*Mentha aquatica* L. decocted extract dos(mg/kg BW/day)			
Males	**0**	**100**	**500**	**1000**
Urea (g/L)	0.44 ± 0.03	0.39 ± 0.02	0.41 ± 0.00	0.48 ± 0.00^b^, *
Creatinine (mg/L)	5.30 ± 0.21	4.90 ± 0.22	5.80 ± 0.10^b^, **	5.00 ± 0.00^c^, *
ALT (units/L)	36.50 ± 1.06	37.50 ± 1.77	33.45 ± 0.32	35 ± 0.00
AST (units/L)	111.50 ± 3.18	100.60 ± 2.12^a^, *	114.50 ± 1.06^b^, **	110.50 ± 0.35^b^, *
Females	**0**	**100**	**500**	**1000**
Urea (g/L)	0.41 ± 0.00	0.38 ± 0.02	0.32 ± 0.11	0.35 ± 0.00
Creatinine (mg/L)	5.60 ± 0.00	4.30 ± 0.39	4.50 ± 0.35	5.25 ± 0.18
ALT (units/L)	44.00 ± 2.83	30.00 ± 1.06	30.50 ± 6.36	47.50 ± 1.06*, ^b^, ^c^
AST (units/L)	144.50 ± 1.77	100.05 ± 2.83^a^, **	121.00 ± 9.00	155.00 ± 7.78^b^, *, **, ^c^

*Note:* Mice (*n* = 5/sex/group) received the decocted extract of *M. aquatica* orally for 28 consecutive days at the following dosages: control group (0 mg/kg, control), G 1 (100 mg/kg), G 2 (500 mg/kg), and G 3 (1000 mg/kg). Hepatic markers were evaluated at the end of the experiments. The obtained result is displayed as the standard deviation ± SEM. Bold values indicate the testeds doses of Ma‐decocted extract.

* *p* < 0.05.

** *p* < 0.01.

^a^ Indicate the variation between the dosages (100,500, 1000) and the control.

^b^ Signify the distinction between doses 100 and (500, 1000).

^c^ Signify variation across (500, 1000).

**TABLE 4:** Hematological parameters in mice orally exposed to *M. aquatica* leaf decocted extract (mg/kg BW).

			Control	Does 100 mg/kg	Does 500 mg/kg	Does 1000 mg/kg
Males	WBC (10^3^ μL^−1^)		10.0 ± 0.00	10.8 ± 0.6	10.0 ± 0.20	10.3 ± 0.20
	RBC (10^6^ μL^−1^)		10.58 ± 0.30	9.0 ± 0.30	10.8 ± 0.10^b^, *	10.0 ± 0.10
	HGB (g/dl)		13.7 ± 0.70	14.0 ± 0.10	16.7 ± 0.2^a^, **, ***, ^b^	15.1 ± 0.10^c^, *
	HCT (%)		45.7 ± 0.10	47.4 ± 0.40	47.5 ± 0.40	50.4 ± 1.20*, **, ^a^, ^b^, ^c^
	MCV (fL)		46.0 ± 0.00	52.4 ± 2.40^a^, *	53.0 ± 0.00^a^, *	50.5 ± 1.80
	MCH (pg)		14.4 ± 0.30	15.5 ± 0.40	15.4 ± 0.30	15.0 ± 0.00
	MCHC (g/dl)		30.5 ± 0.40	29.5 ± 0.40	29.4 ± 0.30	30 ± 0.70
	PLT		1208.5 ± 1.10	1280.5 ± 27.20^a^, *	927.0 ± 0.70***, ^a^, ^b^	1276.5 ± 18.70^c^, ***
	Differential blood count (%)	Neutrophils	6.70 ± 0.14	5.15 ± 1.31	8.80 ± 0.07	4.95 ± 1.80
		Eosinophils	3.40 ± 0.00	2.55 ± 0.18^a^, **	2.90 ± 0.00	2.00 ± 0.21^a^, **, ***, ^c^
		Basophil	0.10 ± 0.02	0.05 ± 0.04	0.10 ± 0.00	0.05 ± 0.04
		Lymphocytes	81.10 ± 0.00	88.80 ± 2.69^a^, *	88.55 ± 0.32^a^, *	92.25 ± 1.94^a^, **
		Monocytes	2.65 ± 0.18	3.45 ± 1.24	0.20 ± 0.00^b^, *	0.75 ± 0.11^b^, *
Females	WBC (10^3^ μL^−1^)		10.50 ± 0.35	9.04 ± 0.51	11.54 ± 2.29	12.26 ± 0.18
	RBC (10^6^ μL^−1^)		10.76 ± 0.16	10.40 ± 0.03	10.91 ± 0.15^b^, *	11.00 ± 0.00^b^, *
	HGB (g/dl)		13.70 ± 0.07	15.60 ± 0.07	15.20 ± 0.92	14.10 ± 2.83
	HCT (%)		45.65 ± 0.11	59.10 ± 2.19	52.20 ± 3.18	46.85 ± 10.15
	MCV (fL)		46.00 ± 0.00	57.00 ± 2.12^a^, **	52.50 ± 2.47	53.00 ± 2.12
	MCH (pg)		14.40 ± 0.28	15.00 ± 0.00	15.00 ± 0.71	16.00 ± 0.00
	MCHC (g/dl)		30.50 ± 0.35	27.80 ± 1.4	29.00 ± 0.00	30.50 ± 0.35
	PLT		2204.50 ± 3.89	1186.00 ± 3.54	1123.00 ± 10.61^a^, *, **, ^b^	1733.00 ± 23.33***, ^a^, ^b^, ^c^
	Differential blood count (%)	Neutrophils	6.70 ± 0.14	5.85 ± 0.88	5.35 ± 0.81	5.60 ± 0.28
		Eosinophils	2.45 ± 0.04	1.9 ± 0.21	1.50 ± 0.07^a^, **	2.10 ± 0.21
		Basophil	0.10 ± 0.00	0.00 ± 0.00	0.05 ± 0.04	0.20 ± 0.07
		Lymphocytes	80.55 ± 0.32	92.60 ± 1.48	75.05 ± 1.80^b^, *	79.30 ± 7.64
		Monocytes	2.40 ± 0.14	1.85 ± 0.39	1.55 ± 1.03	3.00 ± 0.14

*Note:* Results were stated as mean ± S.E.M. White blood cells (WBC), Red blood cells (RBC), Hemoglobin concentration (HGB), mean RBC volume (MCV), mean RBC hemoglobin (MCH), mean RBC hemoglobin concentration (MCHC), Platelets (PLT).

* *p* < 0.05.

** *p* < 0.01.

*** *p* < 0.001.

^a^ Explain the distinction between doses (100, 500, 1000) and control.

^b^ Show the variation between 100 and (500, 1000).

^c^ Indicate the distinction between doses (500 and 1000).

The obtained data showed that the plasma levels of urea and creatinine in male and female mice did not differ substantially from those of the control group (*p* > 0.05). The effect of the decocted extract of *M. aquatica* on hematological markers, notably RBC, HGB, WBC, HCT, MCV, PLT, MCHC, MCH, and differential blood count in both mouse sexes after 28 productive days of oral administration is shown in Table 4. The hematological indices indicate that the decocted extract did not significantly affect most parameters compared to the normal group (control). However, 500 mg/kg of the decocted extract induced a slight increase in HGB and MCV levels (*p* < 0.05 and *p* < 0.001, respectively) for the male treatment group, in contrast to the group under control, whereas a rise in lymphocytes (*p* < 0.05) and a decrease in eosinophil count were observed. Regarding the female‐treated group, no significant changes in hematological parameters were observed, except an increase in MCV (*p* < 0.01). In contrast, a slight decrease in eosinophil counts was observed at a dose of 500 mg/g BW (*p* < 0.01).

#### 3.4.2 Histopathological Evaluation of Animal Organs

**FIGURE 6:**
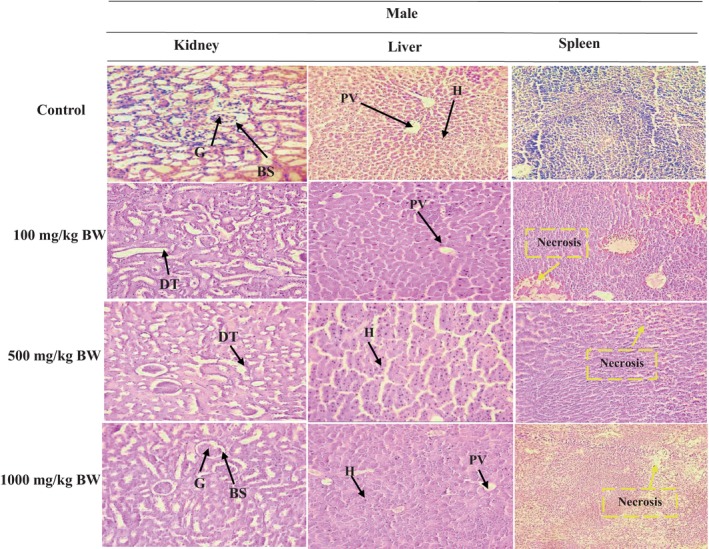
Histopathological examination of the kidney, liver, and spleen tissues of male mice treated with various doses of *M. aquatica* decocted extract (control, 100, 500, and 1000 mg/kg BW), observed under a microscope at 10 × 10X magnification.

**FIGURE 7:**
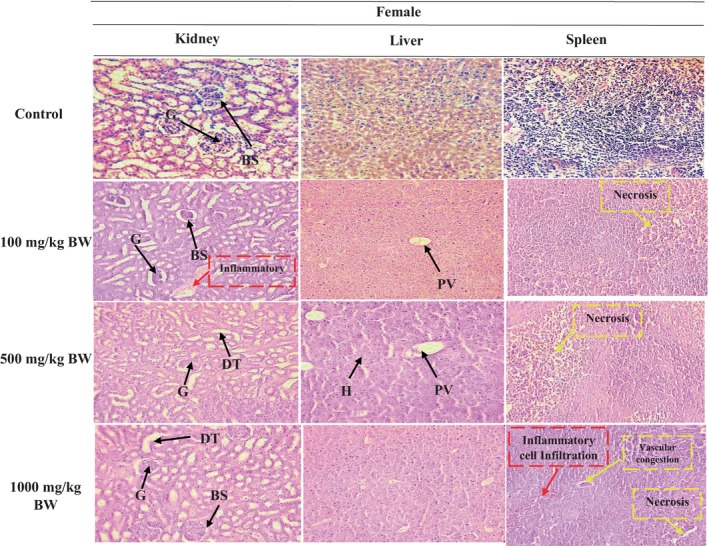
Histopathological examination of the kidney, liver, and spleen tissues of female mice treated with various doses of *M. aquatica* decocted extract (control, 100, 500, and 1000 mg/kg BW), observed under a microscope at 10 × 10× magnification. BS, Bowman space; DT, Distal convoluted tubule; G, Glomerulus; PV, Portal vein; H, Hepatocytes.

Histopathological alterations in the organs treated with the decocted extract of M. aquatica during subacute oral administration were evaluated using hematoxylin–eosin (H&E) staining. The parameters used to track changes in the liver, kidney, and spleen are shown in Figures 6 and 7 and Tables 5, 6, 7.

**TABLE 5:** Intensity of liver damage in different treatment groups.

Markers	Groups			
	Control	100 mg/kg	500 mg/kg	1000 mg/kg
Necrosis	0	0	2	0
Inflammatory cell infiltration	0	0	0	0
Vascular congestion	0	0	0	0
Damaged central vein	0	0	0	0
Damaged hepatocyte cells	0	0	0	0

*Note:* (0) no alteration; (1) Minimal lesions (10% of the affected tissue); (2) Mild lesions (11%–25%); (3) Moderate lesions (26%–50%); (4) Severe lesions.

**TABLE 6:** Intensity of kidney damage in different treatment groups.

Markers	Groups			
	Control	100 mg/kg	500 mg/kg	1000 mg/kg
Lesion/Necrosis	0	0	0	0
Inflammatory lymphocyte infiltration	0	1	0	0
Vascular congestion	0	0	0	0
Glomerular damage	0	0	0	0
Tubular dilatation	0	0	0	0
Renal tubules damage	0	0	0	0

*Note:* (0) no alteration; (1) Minimal lesions (10% of the affected tissue); (2) Mild lesions (11%–25%); (3) Moderate lesions (26%–50%); (4) Severe lesions.

**TABLE 7:** Intensity of spleen damage in different treatment groups.

Markers	Groups			
	Control	100 mg/kg	500 mg/kg	1000 mg/kg
Necrosis	0	1	2	2
Congestion vascular	0	0	0	2
Vascular dilatation	0	0	0	0
Disruption of white‐pulp/red‐pulp	0	0	0	0
Inflammatory cell infiltration	0	0	0	2

*Note:* (0) no alteration; (1) Minimal lesions (10% of the affected tissue); (2) Mild lesions (11%–25%); (3) Moderate lesions (26%–50%); (4) Severe lesions.

As shown in Figures 6 and 7, the histopathological examination of kidney, liver, and spleen sections from the group treated with decocted extract at doses of 100, 500, and 1000 mg/kg bw demonstrated that, compared with the control group, no anomalies were observed in any of the organs. After assessing the vital organs of the treated male (Figure 6) and female mice (Figure 7), a detailed analysis was conducted.

An intact liver structure composed of hepatocytes, the portal triad, and the central vein was observed in the tissue sections for both male and female mice. Hepatocytes were organized into cord‐like structures, with their nuclei and cellular boundaries remaining intact.

Analysis of kidney tissue sections revealed intact glomeruli and renal tubules in both the cortex and medulla for male and female mouse groups, except for a moderate inflammation noticed in the group of females treated with 100 mg/kg bw.

Microscopic observations revealed mid necrosis in the spleen at doses of 100, 500, and 1000 mg/kg bw in males and females, with differences in spleen morphology between the normal and treated groups in females (Table 7). Additionally, moderate vascular congestion, inflammatory cell infiltration, and necrosis were observed in both sexes of the groups exposed to 1000 mg/kg bw of the decocted extract (Figures 6 and 7).

### 3.5 Anti‐Inflammatory Activity

**FIGURE 8:**
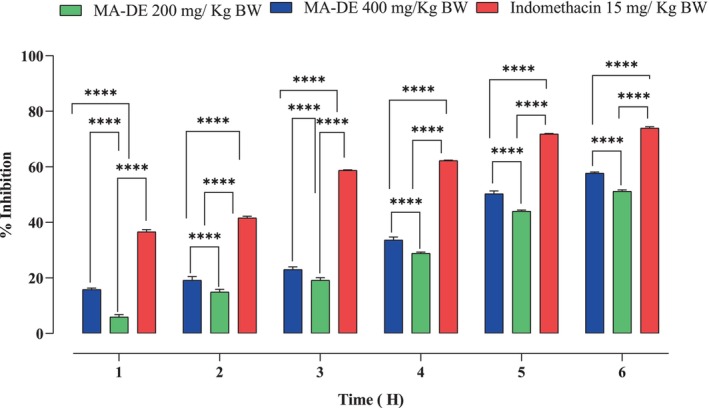
Anti‐inflammatory activity of *M. aquatica* decocted extract at different doses and drugs. Tukey's multiple comparison test relative to the standard: ****p < 0.0001.

As shown in Figure 8, the decocted extract of *M. aquatica* displays marked anti‐inflammatory activity that increases gradually and dose‐dependently over the 6 h of the study period. At all times, the MA‐DE at 400 mg/kg BW yields a greater percent inhibition (57.66%) than the 200 mg/kg BW (51.17%) after 6 h of treatment, suggesting improved activity with increasing dose strength. However, at all times, indomethacin (15 mg/kg) demonstrates the highest inhibitory potential (73.90%), acting as a standard drug. Nevertheless, at a later phase (5–6 h), MA‐DE (400 mg/kg BW) approaches the activity of indomethacin. The data from Tukey's multiple‐comparison tests are significantly different (****p < 0.0001), validating the efficacy of all effects.

### 3.6 Analgesic Activity

#### 3.6.1 Acetic Acid‐Induced Writhing Test

**FIGURE 9:**
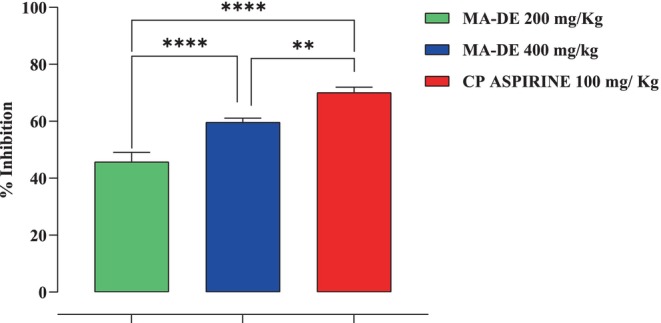
Inhibition of abdominal contortions by different doses of MA‐DE and aspirin. Data were expressed as Mean ± SD (*n* = 5).

The analgesic activity of MA‐DE was assessed using the acetic acid‐induced writhing test, and the results are shown in Figure 9, indicating that MA‐DE inhibits abdominal contortions in a dose‐dependent manner. The lower dose of 200 mg/kg BW causes moderate inhibition (45.72%). In comparison, the higher dose of 400 mg/kg BW results in a substantial increase in analgesic potency, with a percentage increase of 59.62%, reflecting a dose‐dependent increase in efficacy. Aspirin (100 mg/kg), a well‐established drug, shows the highest percentage inhibition (70.05%), demonstrating its effectiveness as an analgesic. From the graphical analysis, it is clear that the treatment groups differ significantly, and the high dose of the compound is substantially more effective than the low dose and the standard drug.

## 4 Discussion

The present study provides the first evidence of the safety profile of Moroccan *M. aquatica* decocted extract following both acute and subacute oral administration in mice. The findings indicate no mortality, behavioral changes, or organ damage in either sex, even at relatively high doses. These results support the potential safe use of this extract in traditional medicine, although caution is advised regarding its intraperitoneal toxicity. Given the high phenolic content, particularly rutin, ferulic acid, and quercetin, the MA‐DE may also offer hepatoprotective and hematological stabilizing effects, which merit further investigation.

Medicinal herbs have long been used in traditional medicine due to their bioactive compounds, which possess anti‐inflammatory, antimicrobial, analgesic, anticancer, and antioxidant properties. However, the safety of these preparations remains a critical concern, especially when consumed without standardized dosing or quality control. Scientific research is crucial for assessing the safety and effectiveness of herbal remedies, identifying potential risks, and establishing guidelines for their use. The current study was conducted to evaluate the potential for acute and subacute oral toxicity of *M. aquatica* , as well as its phenolic content and profile, in an animal model. To our knowledge, this is the first study to reveal the toxic potential of the active phytochemicals in the Moroccan *M. aquatica* decocted extract. Only a limited number of studies have reported on the phytochemicals of *M. aquatica* decocted extract from other countries (Pereira et al. 2012, 2019). The finding of the amount of phenol of *M. aquatica* extracts suggested that the decocted extract contains the highest value with 47.32 ± 0.32 mg GAE/g DW. The value obtained was higher than those reported by Thi et al. (2020), who tested the aqueous extract from *M. aquatica* leaves growing in Vietnam, with a phenolic content of 9.35 ± 1.04 mg GAE/g (Thi et al. 2020), and lower than those reported by (Dorman et al. 2003). The phytochemical profile revealed the presence of fifteen phenolic compounds, notably rutin (14.89 ± 0.03 mg/g), ferulic acid (10.73 ± 0.06 mg/g), and vanillic acid (7.10 ± 0.00 mg/g). The other identified compounds in our extract are gallic acid (1.24 ± 0.15 mg/g), pyrogallol (1.20 ± 0.07 mg/g), tannic acid (1.07 ± 0.03 mg/g), quercetin (1.09 ± 0.11 mg/g), caffeic acid (0.83 ± 0.04 mg/g), and kaempferol (0.41 ± 0.01 mg/g). The high levels of flavonoids and phenolic acids may explain the lack of observed toxicity at therapeutic doses.

Acute oral administration up to 8 g/kg BW caused no deaths or observable toxic symptoms in either sex, placing the extract in the low‐toxicity category according to GHS standards (29). However, intraperitoneal administration revealed an LD_50_ of 5.975 g/kg BW, indicating potential risks when administered via non‐oral routes.

The repeated administration of the *M. aquatica* decocted extract resulted in repeated toxicity consequences (0.1, 0.5, or 1 g/kg BW) for 28 consecutive days in both sexes and did not reveal any indication of intoxication or death. Considerable variations in body mass were observed among the untreated group and the treated groups (0.1, 0.5, or 1 g/kg BW) for both sexes at various time points, with *p* < 0.001. Likewise, there were no substantial differences in the ROW of liver, spleen, or kidney tissue between the sexes during treatment.

Hepatic and renal biomarkers, such as ALT, AST, urea, and creatinine, remained stable across all treatment groups. Since alanine (ALT) is a sensitive indicator of liver injury, its stability reinforces the extract's hepatic safety (Tennant and Center 2008). This could be attributed to the presence of antioxidant compounds such as rutin, quercetin, and ferulic acid. In the same context, previous reports have explored the preventive effect of rutin, quercetin, and kaempferol substances against hepatic damage. The results of Hafez et al. (2015) demonstrate that rutin exhibited a potent hepatoprotective effect, restoring levels of hepatic markers (ALT, AST, ALP) by correcting dysregulation of genes within the IL‐6/STAT3 signaling pathway, leveraging its anti‐apoptotic, anti‐inflammatory, and antioxidant properties (Hafez et al. 2015). Quercetin exhibited its hepatoprotective effect by elevating malondialdehyde (MDA) levels, leading to the formation of advanced oxidation protein products, and by enhancing the activity of antioxidative enzymes (Kasmi et al. 2018). Another study reported that ferulic acid exhibits significant liver‐protective properties through its antioxidative, anti‐inflammatory, and anti‐apoptotic mechanisms (Esmat et al. 2022). Given the detection of rutin, ferulic acid, and quercetin as the major phenolic components in our sample, the observed hepatoprotective potential is highlighted.

Hematological studies provide a clear insight into metabolic irregularities within the body. The blood profile, in particular, often offers a clear and revealing illustration of how the body responds to external challenges, deficiencies, or stress (Seibel et al. 2021). The hematological examination of animals that received a decocted extract of *M. aquatica* revealed no significant alterations in RBC, WBC, HCT, MCH, or MCHC compared with the control group in both sexes, except for slight changes in MCV, PLT, and RDW in male and female mice. Protecting hematological parameters in our mice may be linked to the phenolic‐rich formulation in the *M. aquatica* decocted extract, namely flavonoid molecules. In the same context, previous reports have explored the preventive effects of rutin, quercetin, ferulic acid, and kaempferol on hematological function. An earlier study reported that rutin exerted a potent protective effect on hematological biomarkers by blocking PQ‐provoked hemoglobin oxidation in RBCs (Grinberg et al. 1994). Rutin is also reported to have protective capacity against sideroblastic anemia induced by Isoniazid (INH) by enhancing RBC count, Hb level, and PCV (Abdel‐Ghaffar et al. 2019). Similarly, the data obtained by Vagdevi et al. (2024) indicated that ferulic acid protects cells against haematoxicity induced by folic acid, as evidenced by improvements in Hb concentration, erythrocyte count (TEC), lymphocytes, MCV, and PCV, and a decrease in TLC and neutrophil levels (Vagdevi et al. 2024). Quercetin prevents neutrophilia and lymphopenia induced by acute cadmium administration by reducing inflammatory markers such as TNF‐α and IL‐6 (Donmez et al. 2019). Sex‐specific differences, although not statistically significant, were noted in hematological and biochemical profiles, suggesting a potential hormonal modulation of toxicity pathways that deserves further investigation in future studies.

The prior investigation provides a detailed histopathological assessment of the spleen, kidney, and liver tissues of the treated mice (Figure 6; 7). Thus, histopathological examinations are crucial in exploring this nature as they play a vital role in assessing the safety of consuming certain substances. These tests could confirm or reject the findings of the biochemical and hematological analysis (Everds 2015). Macroscopic and microscopic studies of recovered mouse organs administered low, medium, and high doses consistently demonstrated that all animals treated exhibited undamaged organ tissue, indicating that the 28‐day oral administration of *M. aquatica* decocted extract did not induce any morphological alterations or abnormalities. This integrative approach, combining biochemical, hematological, and histological analyses, provides robust evidence for the short‐term safety of *M. aquatica* decocted extract. However, long‐term exposure, potential reproductive toxicity, and genotoxicity remain to be explored.

Additionally, MA‐DE elicited a dose‐dependent inhibition. However, significant results were obtained with the MA‐DE at a dose of 400 mg/kg BW, with a maximum inhibitory percentage of 57.66% (*p* < 0.0001) over 6 h. These findings surpass those reported by Yousuf et al. (2013), who observed only 42.58% and 45.10% inhibition of paw volume with methanolic *Mentha spicata* extract at 250 and 500 mg/kg, respectively, at the sixth hour (Yousuf et al. 2013). In another study, *Ramex nervosus* demonstrated the highest anti‐inflammatory effect by both the n‐butanol and distilled water extract with inhibition values of 90.64% ± 2.34% and 88.31% ± 2.37%, respectively using 100 mg/mL of plant material (Islam et al. 2025). Similarly, the study by Sajjad et al. (2025) found evidence that phytochemically characterized extracts from *Zingiber officinale* exhibited significant anti‐inflammatory activities suggesting that phytochemicals are potentially biologically active compounds derived from plants (Sajjad et al. 2025).

The aqueous extract also exhibited a promising analgesic effect, as demonstrated by statistically significant (*p* < 0.05) inhibition of abdominal contortions by 59.62% at 400 mg/kg body weight and 45.72% at 200 mg/kg body weight. The results indicate a more pronounced effect as compared to the study by Yousuf et al. (2013), who found that the maximum analgesic effect of the methanolic extract of *M. spicata* (ranging from 40.38% at 250 mg/kg to 42.38% at 500 mg/kg) was produced 3 h after giving the test sample and was similar to the activity of standard Ketorolac (42.73%) (Yousuf et al. 2013).

The anti‐inflammatory and analgesic properties are attributed to the extract's polyphenols. A total of 11 polyphenols were detected, in which rutin, ferulic acid, and vanillic acid were the major ones along with pyrogallol, caffeic acid, gallic acid, coumaric acid, syringic acid, tannic acid, quercetin, and kaempferol (Table 1; Figure 1). Most of these polyphenols exhibit anti‐inflammatory and anti‐hyperalgesemic properties. The rutin and other flavonoids, such as quercetin and kaempferol, modulate major anti‐inflammatory pathways. These work by suppressing the production of pro‐inflammatory cytokines like tumor necrosis factor‐alpha (TNF‐alpha) and Interleukin‐1 beta (IL‐1β), reducing the activation of NF‐κB; thus the expression of enzymes cyclooxygenase 2 or prostaglandin‐endoperoxide synthase 2 (COX‐2; previously known as prostaglandin endoperoxide synthase 2) or inducible nitric oxide synthases (iNOS or inducible nitric oxide synthases) decrease; also by activating the signaling pathways of the antioxidant protein (Alam et al. 2020; Al‐Khayri et al. 2022). Ferulic acid was found to modulate the expression of inflammatory cytokines and pathways, such as NF‐κB, MAPK, and JAK/STAT, as well as to reduce oxidative stress, and this partly mediates its anti‐inflammatory effect (Liu et al. 2025). Furthermore, vanillic acid exerts both anti‐inflammatory and analgesic activities by inhibiting oxidative stress, neutrophil infiltration, and the production of pro‐inflammatory cytokines, as well as by reducing NF‐κB in animal models of inflammatory pain (Calixto‐Campos et al. 2015).

The analgesic property of *M. aquatica* extract is primarily due to its phenolic components. Rutin and quercetin have antioxidant, pro‐inflammatory mediator‐inhibiting, and ion channel‐modulating properties, thereby exerting analgesic effects (Forouzanfar et al. 2025; Patel et al. 2025). Vanillic acid modulates serotonergic and adrenergic mechanisms, as well as TRP and ASIC channels, thereby exerting both central and peripheral analgesic effects (Yrbas et al. 2015). Also, ferulic acid primarily acts through the NO/cGMP/K^+^ channel to produce peripheral analgesia (Kaşık et al. 2019). Other phenolics, such as caffeic, gallic, coumaric, and syringic acids as well as kaempferol, further potentiate analgesia through antioxidant and pro‐inflammatory mediator‐inhibiting effects. The synergistic action of all these compounds would thus produce the pronounced analgesic effect (Kagambega et al. 2022).

## 5 Conclusion

In conclusion, no deaths or adverse effects were observed following acute or subacute oral exposure to the herbal extract of *Mentha aquatica* . There were no significant alterations in hematological or biochemical markers, and no histological patterns were observed in the livers, kidneys, or spleens of male or female mice. The results confirm that the administration doses pose no harm under the tested conditions. In addition, the extract demonstrated strong anti‐inflammatory and analgesic activities, likely attributable to its rich phenolic content. Accordingly, the findings support the safety of *M. aquatica* for potential use in traditional folk medicine.

## Author Contributions

**Mulugeta Tesemma:** writing – original draft, writing – review and editing. **Meryem Tourabi:** conceptualization, writing – original draft, methodology. **Bouchra Louasté:** writing – review and editing, software. **Rafik EL‐Mernissi:** writing – original draft, methodology. **Karima El‐Yagoubi:** data curation, writing – original draft. **Ohoud A. Alghamdi:** supervision, investigation. **Layla Tahiri Elousrouti:** formal analysis, writing – review and editing. **Musa A. Said:** resources. **Khaoula Faiz:** methodology, writing – original draft. **Abdel‐Rhman Z. Gaafar:** investigation, supervision. **Elhoussine Derwich:** data curation, formal analysis. **Esmael M. Alyami:** supervision, investigation. **Badiaa Lyoussi:** writing – original draft, writing – review and editing. **Mohammed Merzouki:** software, data curation. **Hina Ali:** resources.

## Funding

This work is financially supported by the Ongoing Research Funding Program (ORF‐2026‐686), King Saud University, Riyadh, Saudi Arabia.

## Disclosure

*ARRIVE guidelines*: The experimentation was conducted according to ARRIVE guidelines. *Plant collection approval*: No approval is needed from the authority in Morocco to collect *Mentha aquatica* L. for research purposes. *IUCN Policy statement*: The collection of plant material complies with relevant institutional, national, and international guidelines and legislation.

## Ethics Statement

The ethical committee of Sidi Mohamed Ben Abdellah University, Fez, Morocco, revised and approved this work under N°L.20.USMBA‐SNAMOPEQ 2020‐03. Notably, all animal experimentation was conducted in accordance with applicable laws, regulations, and guidelines, prioritizing animal welfare and minimizing potential harm.

## Consent

The authors have nothing to report.

## Conflicts of Interest

The authors declare no conflicts of interest.

## Data Availability

All data generated or analyzed during this study are included in this published article.
